# The use of serum deoxythymidine kinase as a prognostic marker, and in the monitoring of patients with non-Hodgkin's lymphoma.

**DOI:** 10.1038/bjc.1983.78

**Published:** 1983-04

**Authors:** J. S. Gronowitz, H. Hagberg, C. F. Källander, B. Simonsson

## Abstract

A recently developed enzyme assay, utilizing [125I]-iododeoxyuridine as substrate, and capable of detecting normal levels of serum deoxythymidine kinase (s-dTk), was used in an investigation of sera from 155 untreated patients with non-Hodgkin's lymphoma (NHL). The patients were classified at the discovery of disease, both according to spread (stages I-IV according to the Ann Arbor classification) and to tumour histology (the Kiel classification). The results showed a significant correlation between s-dTk level and the extent of disease, as well as to the malignancy; i.e. the more advanced the disease or the more aggressive the tumour, the higher the s-dTk values. Greater than 100-fold increases in s-dTk levels were found in some patients compared to those reported for healthy individuals. A high pretreatment level of s-dTk for patients in stages III-IV correlated with a poor prognosis for the patient in terms of survival. This was consistent even when only patients in stages III-IV with "high-grade" malignant lymphomas were included in the analysis. Longitudinal studies of s-dTk levels in 19 NHL patients showed that s-dTk increases with progression of the disease, decreases during successful therapy, and finally increases during relapse. It is concluded that s-dTk could be used both as a prognostic marker and to monitor the effect of therapy in NHL patients.


Br. J. Cancer (1983), 47, 487–495

### The use of serum deoxythymidine kinase as a prognostic marker, and in the monitoring of patients with non-Hodgkin's lymphoma

J.S. Gronowitz, H. Hagberg[1], C.F.R. Källander & B. Simonsson

*Department of Medical Virology, Biomedical Center, Uppsala University, S-751 23 Uppsala, [1]Department of Internal Medicine, University Hospital, Uppsala, Sweden.*

**Summary** A recently developed enzyme assay, utilizing [$^{125}$I]-iododeoxyuridine as substrate, and capable of detecting normal levels of serum deoxythymidine kinase (s-dTk), was used in an investigation of sera from 155 untreated patients with non-Hodgkin's lymphoma (NHL). The patients were classified at the discovery of disease, both according to spread (stages I-IV according to the Ann Arbor classification) and to tumour histology (the Kiel classification). The results showed a significant correlation between s-dTk level and the extent of disease, as well as to the malignancy; i.e. the more advanced the disease or the more aggressive the tumour, the higher the s-dTk values. Greater than 100-fold increases in s-dTk levels were found in some patients compared to those reported for healthy individuals. A high pretreatment level of s-dTk for patients in stages III-IV correlated with a poor prognosis for the patient in terms of survival. This was consistent even when only patients in stages III-IV with "high-grade" malignant lymphomas were included in the analysis. Longitudinal studies of s-dTk levels in 19 NHL patients showed that s-dTk increases with progression of the disease, decreases during successful therapy, and finally increases during relapse. It is concluded that s-dTk could be used both as a prognostic marker and to monitor the effect of therapy in NHL patients.

Three different isoenzymes of deoxythymidine kinase (dTk) (ATP:thymidine 5'-phosphotransferase (EC 2.7.1.21)) have been found in human cells (Kit; 1979). One of these, dTK-F, the cytosolar dTk, occurs in high amounts in dividing cells (stages G1→S) and is more or less absent in resting differentiated cells (Bello, 1974). For this reason dTk activity has been studied in relation to tumor cell growth, employing different animal systems (Bresnick *et al.*, 1969, 1971, Rothschild & Black; 1970, 1973). Studies of 23 matched human neoplastic and normal tissue pairs showed higher dTk activity in tumours, except for bronchogenic carcinomas and hypernephromas (Gordon *et al.*, 1968). Recent reports have demonstrated enhanced dTk-F levels in peripheral blood lymphocytes of some patients suffering from non-Hodgkin's lymphoma (NHL) or active chronic lymphocytic leukaemia (Ellims *et al.*, 1981*a*; Ellims, 1981; Kreis *et al.*, 1982). These investigators, although using an enzyme assay with radiolabelled dT as substrate, were able to find dTk-F in the serum of 26 patients with advanced NHL. By comparing these patients with other patients having mainly dTk-A (the mitochondrial isoenzyme) levels a significant difference in median survival time was found i.e. dTk-F positive patients had a short survival time (Ellims *et al.*, 1981*b*).

An optimized dTk assay suitable for the detection of viral dTk isoenzymes based on the use of [$^{125}$I]-iododeoxyuridine (IUdR) as the substrate has been presented (Gronowitz & Källander; 1980). A different assay system, still based on the use of [$^{125}$I]-IUdR, as the substrate, optimized to measure dTk-F was recently developed (Gronowitz *et al.*, submitted). By the use of this method normal serum-dTk levels (s-dTk) could be measured and transiently enhanced s-dTk levels were found during acute-convalescent stage of infections with morbilli, rubella and different herpesviruses. Elevated s-dTk levels, were also found to be characteristic of pernicious anaemia and of different malignancies, such as chronic granulocytic leukaemia, acute myelocytic and lymphocytic leukaemia, small cell lung cancer, and non-Hodgkin's lymphoma (NHL) (Gronowitz *et al.*, submitted).

The aim of the present study was to correlate s-dTk levels of patients with NHL to other parameters, such as spread, grade of malignancy,

Correspondence: Dr. Hans Hagberg, Department of Medicine, Akademiska sjukhuset, S-750 14 Uppsala, Sweden.

Received 9 November 1982; accepted 15 January 1983

© The Macmillan Press Ltd., 1983

and to therapic effects, in order to evaluate s-dTK as a prognostic and diagnostic serum marker.

### Materials and methods

*Enzyme assay*

The assay utilizes [$^{125}$I]-IUdR (final concentration $10^{-7}$ M, 130–160 Ci mM)$^{-1}$, as substrate, and is described in detail elsewhere (Gronowitz *et al.*, submitted). This new assay system gives a linear turnover of substrate for the cellular dTk-F for more than 2 h. In order to minimize fluctuations in the assay, a biological control for the isotope was utilized, and all values were recalculated to units (Gronowitz *et al.*, submitted). Under the conditions used 1 unit of enzyme is equal to an enzyme activity of 1.2 $10^{-18}$ katal, and gives ~1000 cpm, with the amount of isotope used. The average s-dTk level in healthy subjects is estimated to be 2.5 units $\mu l^{-1}$ (s.d. 1.25) (Gronowitz *et al.*, submitted). All units given are calculated per $\mu$l serum.

*Serum sampling*

Blood samples were collected from 155 untreated patients, diagnosed to have NHL, who were referred to the University Hospital in Uppsala between February 1979 and September 1981. The mean age of the patients was 54 y (range, 16–83 y). Sera, from patients receiving intermittent therapy, used in longitudinal studies of s-dTk were collected before chemotherapy. All sera were stored at −20°C until analyzed.

*Classification and staging of patients*

Histopathological classification was made by Dr. C. Sundström, Department of Pathology, Uppsala according to the Kiel classification system (Gerard-Marchant *et al.,* 1974). In the original classification there are 2 prognostic subgroups, but recently 3 prognostic subgroups were recognized in the region studied (Glimelius & Sundström; 1982). These results have been applied in the present investigation. The distribution of the patients is shown in Table I, which also gives the different histological types of each group and their abbreviations. The criteria for staging adopted at the Ann Arbor symposium on Hodgkin's disease was used (Carbone *et al.,* 1971). The initial evaluation included physical examination, complete blood counts, urine analysis, measurement of the erythrocyte sedimentation rate and serum concentrations of uric acid, liver enzymes, and bilirubin. Both sternal bone marrow aspiration and iliac crest core biopsy were performed. Radiological examinations comprised chest X-ray, and computerized tomography of the abdomen. Further ultrasound scanning of the abdomen was performed. Lymphangiography was performed only in a few patients. Restaging was performed after 6–8 months of treatment. All examinations were repeated if necessary, except lymphangiography. Complete remission was defined as total disappearance of the tumour for at least one month, partial remission as regression of tumour mass by >50%, and progressive disease as tumour progression by >25%.

**Table I** Distribution of the histological subgroups according to the Kiel classification and the stage of disease.

| | *Abbreviation* | *Stage: I* | *II* | *III* | *IV* |
|---|---|---|---|---|---|
| "High-grade" malignancy | | | | | |
| Lymphoblastic | (LB) | 1 | 1 | 0 | 3 |
| Immunoblastic | (IB) | 2 | 2 | 1 | 3 |
| Centroblastic | (CB) | 13 | 3 | 2 | 21 |
| Diffuse centroblastic-centrocytic | (D CB−CC) | 4 | 1 | 2 | 6 |
| "Intermediate-grade" malignancy | | | | | |
| Immunocytic | (IC) | 9 | 3 | 0 | 21 |
| Centrocytic | (CC) | 0 | 0 | 0 | 1 |
| Follicular and diffuse centroblastic-centrocytic | (F+D CB−CC) | 1 | 1 | 2 | 7 |
| "Low-grade" malignancy | | | | | |
| Lymphocytic | (LC) | 0 | 0 | 0 | 6 |
| Follicular centroblastic-centrocytic | (F CB−CC) | 8 | 2 | 6 | 11 |
| Unclassified | | 3 | 0 | 1 | 8 |
| Totals | | 41 | 13 | 14 | 87 |

*Treatment of patients*

All 54 patients in stage I and II (see Table I) were irradiated with 40–45 Gy. Eight of these patients took part in a separate clinical study and received adjuvant chemotherapy with COP (cyclophosphamide, vincristine, prednisone) as described by Bagley *et al.* (1972). The 101 patients in stages III and IV (see Table I, for abbreviations) were treated according to the following principles: patients with CB, LB and IB were randomized and treated either with CHOP (COP+doxorubicin), as described by McKelvey *et al.* (1976), or MEV (methotrexate, cyclophosphamide, vincristine), as described by Lauria *et al.* (1978). All patients with D CB–CC, F+D CB–CC, IC and patients with LC or F CB–CC, who had progressive disease, were randomized and treated with either COP or intermittent Prednimustine (a chloroambucil ester of prednisolone, AB Leo, Sweden), 150–200 mg daily for five days every fortnight. Patients were treated with MEV or CHOP for 6 months, then restaged, and if complete remission was found, treatment was omitted. Patients in complete remission after treatment with Prednimustine or COP received maintenance treatment at successively prolonged intervals for up to 2.5 y. Patients in partial remission, progressive disease or relapse received individual treatment. All patients could be followed from admission to death or completion of follow-up.

*Statistical methods*

The calculations of survival and the statistical significance test (log rank test) were carried out according to the method of Peto *et al.* (1977).

### Results

*Pretreatment s-dTk level in relation to stage*

The distribution of pretreatment s-dTk levels, in relation to stage of disease, is presented in Figure 1. The mean pretreatment s-dTk value in stages I-II was 4.7 units and in stages III-IV, 32.0 units. The difference is highly significant ($P<0.001$).

*Pretreatment s-dTk level in relation to histology*

Pretreatment s-dTk levels found in patients of stages III-IV distributed according to grade of malignancy, are illustrated in Figure 2. The results show that s-dTk level correlates with malignancy, and a mean value of 4.6 units was found for patients with "low-grade" malignancy, compared to

**Figure 1** s-dTk in 155 untreated patients with NHL correlated to stage of the disease.

**Figure 2** s-dTk in 101 untreated NHL patients in stages III-IV divided into three grades of malignancy.

28.8 units for the "intermediate-grade" and 45.0 units for the "high-grade" group. The differences between each group is significant at the $P < 0.01$ level. Among the stage I-II patients with s-dTk >5 units (Figure 1), 6/8 had "high-grade" and the other 2 "intermediate grade" malignancies.

*Pretreatment s-dTk level in relation to survival*

A discriminant analysis of pretreatment s-dTk levels for patients in stages III-IV, in relation to survival time was carried out. The results showed that the best discriminating level was 10 units, giving a highly significant difference in survival time ($P < 0.001$) between the 2 groups. The patients with the low s-dTk values had an actuarial survival of 85% at the 32nd month compared to <30% for those with levels above 10 units (Figure 3a). As it is well known that the grade of malignancy influences survival, we also compared s-dTK levels with survival time for patients with "high grade" malignancy in stages III-IV. A significant difference was still found ($P < 0.02$), with patients having pretreatment s-dTk >10 units dying sooner (Figure 3*b*).

*Pretreatment s-dTk level of stage I-II patients in relation to relapse*

Of the patients in stage I and II, 8 received adjuvant chemotherapy besides local radiotherapy, and the remaining 46 local radiotherapy only. In the latter group 8 had pretreatment s-dTk levels >5 units, and 6 of them relapsed within a year. In contrast, only 8 out of the other 38 patients, had a recurrence within the same time. The 2 patients with the highest s-dTk levels, 45.4 and 23.3 units respectively, rapidly sustained systemic spread during initial radiotherapy, indicating an underestimation of the staging.

*S-dTk level in longitudinal studies of NHL patients related to treatment and development of disease.*

S-dTk was measured during the treatment of NHL patients. For intermittently-treated patients the sera were collected before therapy at each time point. Representative examples of variations in s-dTk are given for 19 patients in Figures 4–7, and the first value always represents pretreatment s-dTk. Clinical data for the 19 patients, such as age, sex, tumour,

**Figure 3** (a) probability of survival for 101 NHL patients in stages III-IV with pretreatment s-dTk <10 units (○–○; n=50), and >10 units (●–●; n=51). (Number in parenthesis indicates remaining individuals at observation after 32 months). (b) Probability of survival for 38 NHL patients in stages III-IV with "high grade" neoplasms. Pretreatment level of s-dTk<10 units (○–○; n=11), and >10 units (●–●; n=27). (Number in parenthesis indicates remaining individuals at observation after 32 months).

**Table II** Clinical data from 19 NHL patients who were followed longitudinally with serial measurements of s-dTk.

| *Figure* | *Patient* | *Sex/Age* | *Histology* | *Stage* | *Initial treatment* | *Response to initial treatment* | *Relapse treatment* |
|---|---|---|---|---|---|---|---|
| 5 | BB | M 36 | F+D CB−CC | IV | Splenectomy | | |
| 5 | AJ | F 77 | IC | IV | Splenectomy | | |
| 6 | KA | F 31 | IB | I | RT | CR | MEV |
| 6 | MO | M 70 | IC | II | RT | CR | Prednimustine |
| 6 | HH | M 68 | LB | II | RT | CR | CHOP |
| 7a | LJ | F 77 | IB | IV | COP | PD | |
| 7a | AM | F 61 | D CB−CC | IV | Prednimustine+COP | PD | |
| 7a | AT | F 61 | CB | IV | CHOP | PD | |
| 7b | CJ | F 50 | CC | IV | COP | PR | |
| 7b | KL | M 71 | F CB−CC | IV | COP | PR | |
| 7c | LD | F 55 | F CB−CC | IV | Prednimustine | CR | |
| 7c | KE | F 60 | CB | IV | CHOP | CR | |
| 7c | LH | F 74 | CB | III | MEV | CR | |
| 7c | GT | M 63 | IB | IV | MEV | CR | |
| 8a | OE | M 55 | CB | IV | MEV+CHOP | PD | |
| 8a | EM | M 62 | Unclass. | IV | Prednimustine+COP | PD | |
| 8b | AH | M 36 | CB | IV | CHOP | PR | COMLA |
| 8b | EL | F 69 | Unclass. | IV | COP | PR | CHOP |
| 8b | GE | M 68 | F+D CB−CC | IV | COP | CR | CHOP |

*Abbreviations*

COP = Cyclophosphamide, Vincristine, Prednisone

MEV = Methotrexate, Cyclophosphamide, Vincristine

CHOP = Cyclophosphamide, Adriamycine, Vincristine, Prednisone

COMLA = Cyclophosphamide, Vincristine, Methotrexate with leucovorin rescue, Cytarabine

Prednimustine = a chlorambucil ester of prednisone

RT = Radiotherapy

CR = Complete remission

PR = Partial remission

SD = Stationary disease

PD = Progressive disease

F = Follicular

D = Diffuse

CB = Centroblastic

IB = Immunoblastic

CC = Centrocytic histology, stage on admission, initial treatment, response to treatment and relapse therapy, are summarized in Table II. Figure 4 shows that a rapid fall in s-dTk was observed after splenectomy as examplified in 2 patients. Three patients in stage I or II with low s-dTk, treated initially only with local radiotherapy and having elevated s-dTk at relapse are shown in Figure 5. All 3 received chemotherapy during relapse, and in two of them s-dTk was measured after chemotherapy was given. Patient MO responded to the therapy and s-dTk was found to decrease, whereas HH did not respond and s-dTk remained unchanged.

The remaining 14 patients were all in stage III or IV when therapy was started. Five of them did not respond to the initial treatment, and, 3 died without any improvement (Figure 6a); as seen in Figures 6a and 7a, progression of the disease was accompanied by increasing s-dTk. The other 2 responded and underwent temporary improvement when given a different therapy; s-dTk mirrored this clinical course by a temporary decrease followed by continued increase (Fig. 7a).

Four patients were considered to be in partial remission after 6 months of treatment, and their s-dTk levels were found to decrease, but not to normality (Figure 6b, and 2 patients in Figure 7b).

Two of these relapsed and increasing s-dTk levels ensued (Figure 7b).

Finally, 5 patients went into complete remission, which was followed by normalization of s-dTk (Figure 6c, and patient GE in Figure 7b). One of them relapsed (Figure 7b) and a high s-dTk level was found.

**Figure 4** s-dTk in two NHL patients before and within a week after splenectomy.

**Figure 5** s-dTk in 3 patients with initial localized NHL (stages I and II), who later relapsed. 1). Before local radiotherapy 2). After local radiotherapy 3). At systemic relapse 4). After the first treatment with chemotherapy. M.O. responded, whereas H.H. did not.

**Figure 6** s-dTk in NHL patients. (a) patients with progressive disease, (b) two patients treated to partial remission, (c) four patients treated to complete remission.

**Figure 7** s-dTk followed longitudinally in patients, where both progressive disease (a), and remission (b) occur, (**A**) two patients with progressive disease, who after change of therapy were treated to partial remission before the disease progressed again, (**B**) three patients treated to remission (A.H. and E.L. to partial, and G.E. to complete remission). All of them later relapsed. A.H. responded transiently to further therapy.

### Discussion

Despite information on histopathology, stage and other clinical parameters it is often difficult to predict the clinical course of lymphomas of the non-Hodgkin's type. There is therefore a need for a multiparametric approach to define subgroups of patients with different clinical behaviour. Among biochemical serum markers, the most important of those previously reported are $\beta_2$-microglobulin (Child *et al.*, 1980, Hagberg *et al.*, In press) and lactodehydrogenase (Ferraris *et al.*, 1979, Schneider *et al.*, 1980).

By a recently developed method for quantification of s-dTk in normal serum, variations in s-dTk in NHL patients were detected. The results showed that s-dTk levels in pretreatment sera correlated with the spread of disease, i.e. most patients in stages I and II had normal values, whereas elevated levels, in some cases $>100\times$ the normal value, could be found in sera of patients in stages III and IV. When patients in stages III-IV were divided according to the malignancy of the tumour, it was found that high s-dTk levels, correlated with high malignancy. Discriminant analysis of pretreatment s-dTk levels of patients in stages III-IV, relative to survival time, revealed two groups which differed significantly in survival time. The optimal discrimination level of s-dTk was found to be 10 units, with the significant longer survival associated with those having values $<10$ units. However it could be argued that patients in stages III and IV comprise those with tumours of low malignancy and others with "high grade" malignant tumours. For this reason sera were analysed from a group of patients in stages III and IV who all had "high grade" malignant tumours. The results still showed significantly different survival times for those having s-dTk $<10$ units compared to those having s-dTk $>10$ units. Moreover, for stage I-II patients, who received only radiotherapy, a higher relapse frequency was found during the first year when the pretreatment s-dTk level was $>5$ units. The method may thus be able to identify those patients for whom local radiotherapy cannot be considered sufficient. From the results discussed above it was concluded that pretreatment s-dTk values provide a useful prognostic tool. The prognostic value of s-dTk found in this study is in agreement with a previous smaller study which used another method to determine s-dTk (Ellims *et al.*, 1981*b*).

Longitudinal studies of patients with low or high pretreatment values showed that the s-dTk level reflected the activity of the disease; i.e. the titres decreased during tumour regression and increased during tumour progression. For this reason s-dTk can be used to detect relapses. Patients with therapy-resistant or only partially-responsive disease had a persistent elevation of s-dTk. Since sera were always collected before intermittent therapy was administered the long term effects of treatment were mirrored in the s-dTk level. However, the short- to immediate-term effect of successful chemotherapy on s-dTk, as indicated in preliminary studies, is a transient rise (data not shown). The utilization of dTk release as a direct marker for therapic efficacy has been suggested previous from cell cultures studies (Kessel & Wodinsky; 1970, Taylor *et al.*, 1981).

The origin of the elevated s-dTk is not yet known. We have indicated elsewhere that the serum enzyme is of cellular origin, and probably related to the cytosolar dTk-F (Gronowitz *et al.*, In press). The enzyme is most likely released into the serum upon the death of cells in a proliferating stage, which seems to be a rare event in healthy individuals. However, the s-dTk level can be increased due to virus infections and pernicious anaemia, but s-dTk is not a marker for non-specific liver damage (Gronowitz *et al.*, In press). This may, however, be a rare complication since most viruses which are known to give elevated s-dTk, are confined to childhood.

The huge variation in s-dTk found in pretreatment sera of NHL patients considered to have the same clinical picture, could be due to different amounts of dTk per cell in individual tumours or to a higher turnover of the tumour cells. This has to be investigated, as well as how s-dTk expression relates to other better known markers, such as $\beta_2$-microglobulin and lactodehydrogenase (studies in progress).

The findings reported in this communication show that s-dTk is a prognostic tool and a valuable marker in monitoring the course of the disease in NHL patients. Further, the simplicity of the s-dTk assay makes it suitable for widespread clinical use.

We are indebted to Dr. U. Pettersson for valuable discussions and critical review of the manuscript. U. Långström–Persson, E. Nilsson and K. Hjelmar provided excellent technical assistance.

### References

BAGLEY, C.M., De VITA, V.T. & BERARD, C.W. (1972). Advanced lymphosarcoma intensive cyclical combination chemotherapy with cyclophosphamide, vincristine and prednisone. *Ann, Intern, Med.*, **76**, 227.

BELLO, L.J. (1974). Regulation of thymidine kinase in human cells. *Exp. Cell Res.*, **89**, 263.

BRESNICK, E, MAINIGI, K.D, MAYFIELD E.D. Jr & MORRIS H.P. (1969). Activities of enzymes of pyrimidinenucleotide synthesis in slowly kidney tumors. *Cancer Res.*, **29**, 1932.

BRESNICK, E, MAYFIELD, E.D., Jr, LIEBELT A.G. & LIEBELT R.A. (1971). Enzyme patterns in a group of transplantable mouse hepatomas of different growth rate. *Cancer Res.*, **31**, 743.

CARBONE, P.P., KAPLAN, H.S., MUSSHOFF, K, SMITHERS, D.W. & TUBIANA, M. (1971). Report of the Committee on Hodgkin's disease staging classification. *Cancer Res.*, **31**, 1860.

CHILD, J.A., SPÄTI, B., ILLINGSWORTH, S, & 5 others. (1980). Serum $\beta_2$-microglobulin and C-reactive protein in the monitoring of lymphomas. *Cancer.* **45**, 318.

ELLIMS, P.H., VAN DER WEYDEN, M.B. & MEDLEY, G. (1981*a*). Thymidine kinase isoenzymes in malignant lymphoma. *Cancer Res.*, **41**, 691–5.

ELLIMS, P.H. (1981). Thymidine kinase isoenzymes in chronic lymphocytic leukaemia. *BR. J. Haematol.*, **49**, 479.

ELLIMS, P.H., GAN, T.E., MEDLEY, G. & VAN DER WEYDEN, M.B. (1981*b*). Prognostic relevance of thymidine kinase isozymes in adult non-Hodgkin's lymphoma. *Blood.* **58**, 926.

FERRARIS, A.M., GIUNTINI, P., GAETANI, G.F. (1979). Serum lactic dehydrogenase as a prognostic tool for non-Hodgkin lymphomas. *Blood*, **54**, 928–932.

GERARD-MARCHANT R., HAMLIN, I., LENNERT, K., RILKE, F., STANSFELD, A.F. & VAN UNNIK, J.A.M. (1974). Classification of non-Hodgkin's lymphomas. *Lancet ii.*, 406.

GLIMELIUS, B. & SUNDSTRÖM, C. (1983). Morphological classification of non-Hodgkin's lymphoma I. A retrospective analysis using the Kiel classification. *Acta Radiol. Oncol*, (In press).

GORDON, H.L., BARDOS, T.L., CHMIELEWICZ, Z.F. & AMBRUS, J.L. (1968). Comparative study of the thymidine kinase and thymidylate kinase activities and of feed back inhibition of thymidine kinase in normal and neoplastic tissue. *Cancer Res.*, **28**, 2068.

GRONOWITZ, J.S. & KÄLLANDER, C.F.R. (1980). Optimized assay for thymidine kinase and its application for the detection of antibodies against Herpes simplex virus type 1- and 2-induced thymidine kinase. *Infec, Imm.*, **29**, 425.

GRONOWITZ, J.S., KÄLLANDER, C.F.R., DIDERHOLM, H., PETTERSSON, U. Deoxythymidine kinase, a novel serum marker of viral and tumor disease (submitted for publication).

HAGBERG, H., KILLANDER, A. & SIMONSSON, B. (1983). Serum $\beta_2$-microglobulin in malignant lymphoma. *Cancer* (in press).

KESSEL, D, WODINSKY, I. (1970). Thymidine kinase as a determinant of the response to 5-fluoro-2-deoxyuridine in transplantable murine leukemias. *Mol. Pharmacol.*, **6**, 251.

KIT, S. (1979). Viral-associated and induced enzymes. *Pharmacol Therapeut.*, **4**, 501.

KREIS, W., ARLIN, Z., YAGODA, A., LEYLAND-JONES, B.R., FIORI, L. (1982). Deoxycytidine and deoxythymidinekinase activity in plasma of mice and patients with neoplastic disease. *Cancer Res.*, **42**, 2514.

LAURIA, F., BACCARANI, M., FIACCHINI, M., GOBBI, M. TURA, S. (1978). Methotrexate, cyclophosphamide and vincristine (MEV regimen) for non-Hodgkin's lymphoma. *Cancer Treat. Rep.*, **63**, 1193.

McKELVEY, E.M., GOTTLIEB, J.A., NILSON, H.E. & 10 others. (1976). Hydroxyldaunomycin (Adriamycin) combination chemotherapy in malignant lymphoma. *Cancer*. **38**, 1484.

PETO, R., PIKE, M.C., ARMITAGE, P. & 6 others. (1977). Design and analysis of randomized clinical trials requiring prolonged observation of each patient. *Br. J. Cancer.*, **35**, 1.

ROTHSCHILD, H. & BLACK, P.H., (1970). Effects of loss of thymidine kinase activity on the tumorigenicity of clones of SV40-transformed hamster cells. *Proc. Natl. Acad. Sci.*, **67**, 1042.

ROTHSCHILD, H. & BLACK, P.H., (1973). Investigations of the mechanism of decreased tumorigenicity of cells grown in BrdU. *J. Cell Physiol.*, **81**, 217.

SCHNEIDER, R.J., SEIBERT, K., PASSE, S. & 5 others. (1980). Prognostic significance of serum lactate dehydrogenase in malignant lymphoma. *Cancer.*, **46**, 139.

TAYLOR, A. Jr., JONES, O.W., & GRISHAVER, M.A. (1981). Effect of 5-fluorouracil on the release of thymidine kinase from hepatoma cells *in vitro*. *Cancer Res.*, **41**, 192.


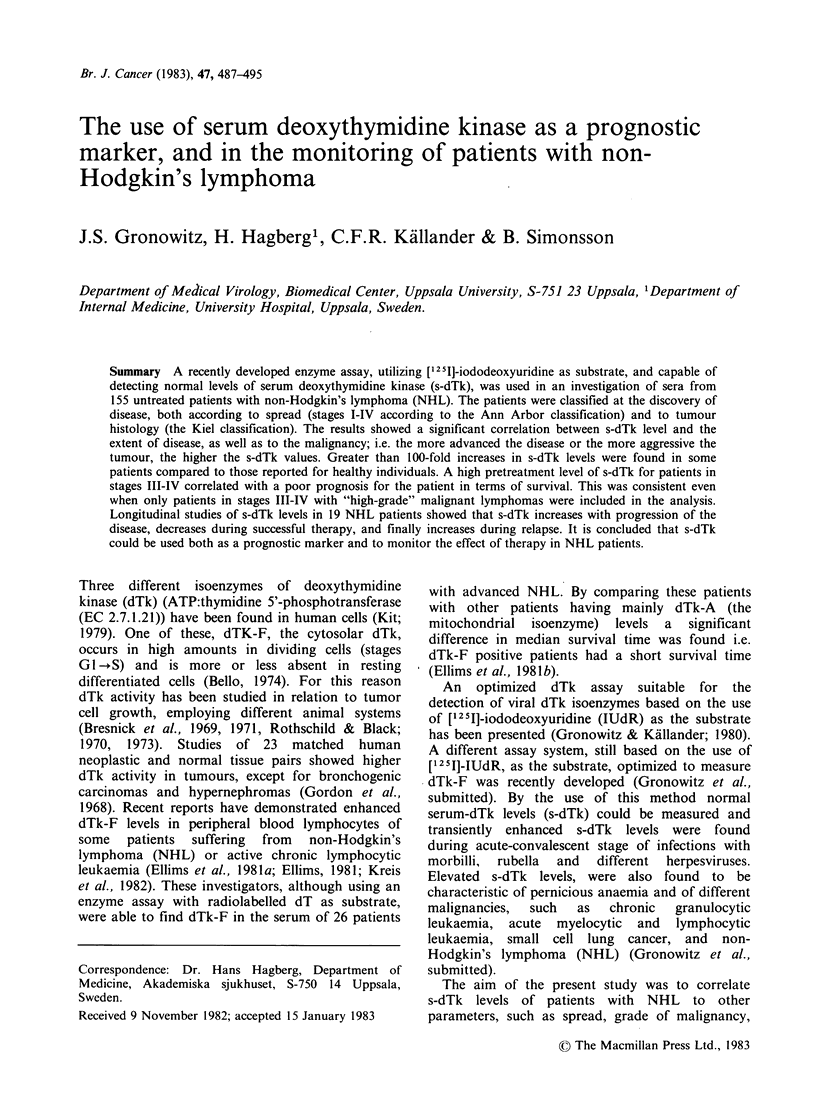

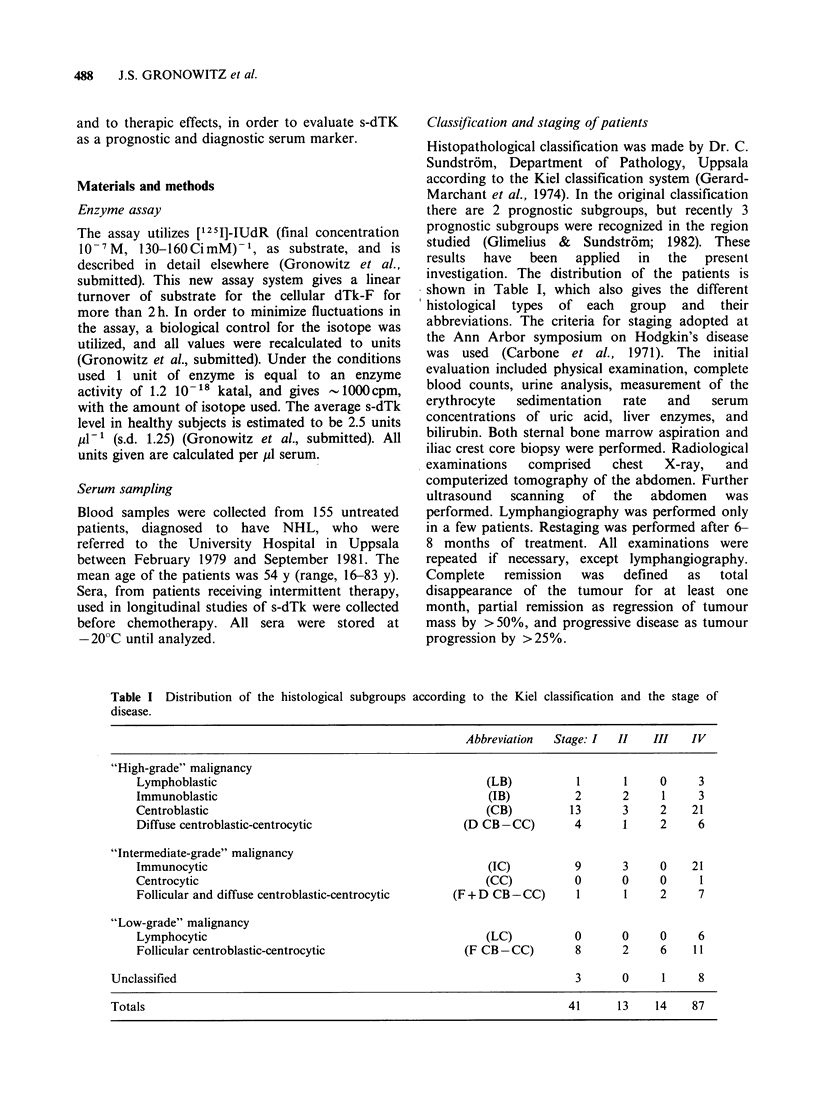

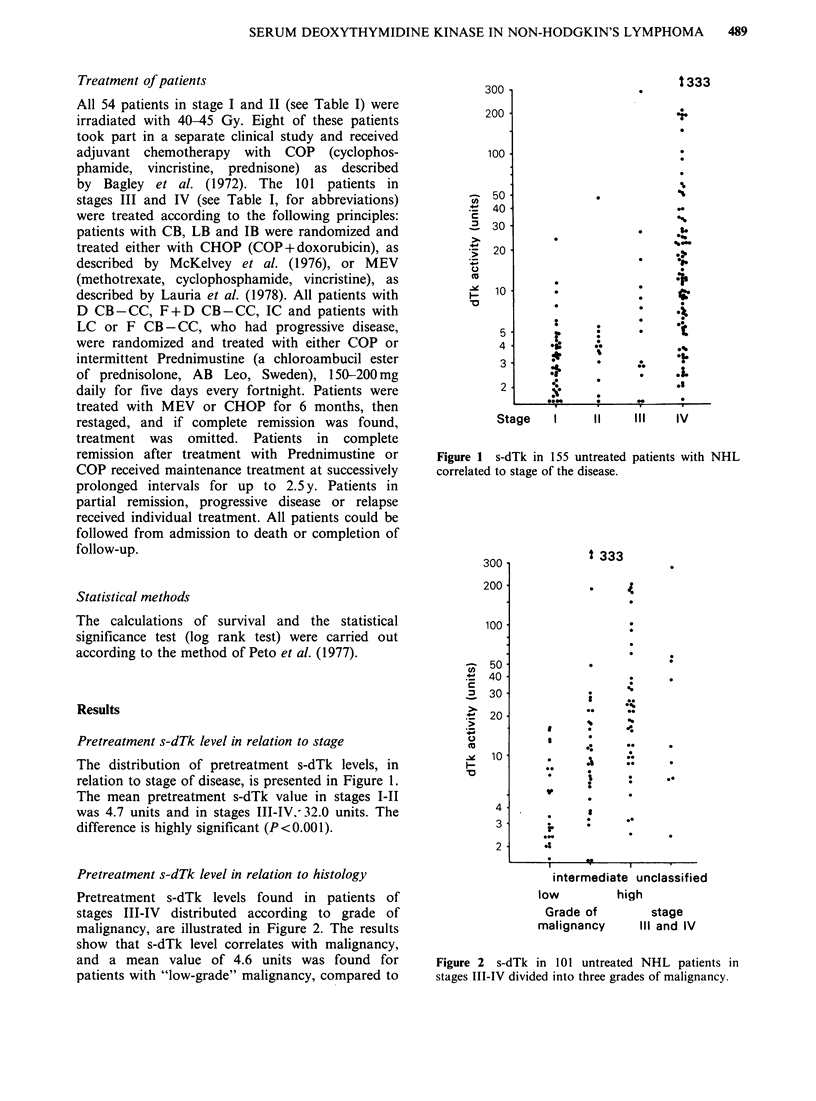

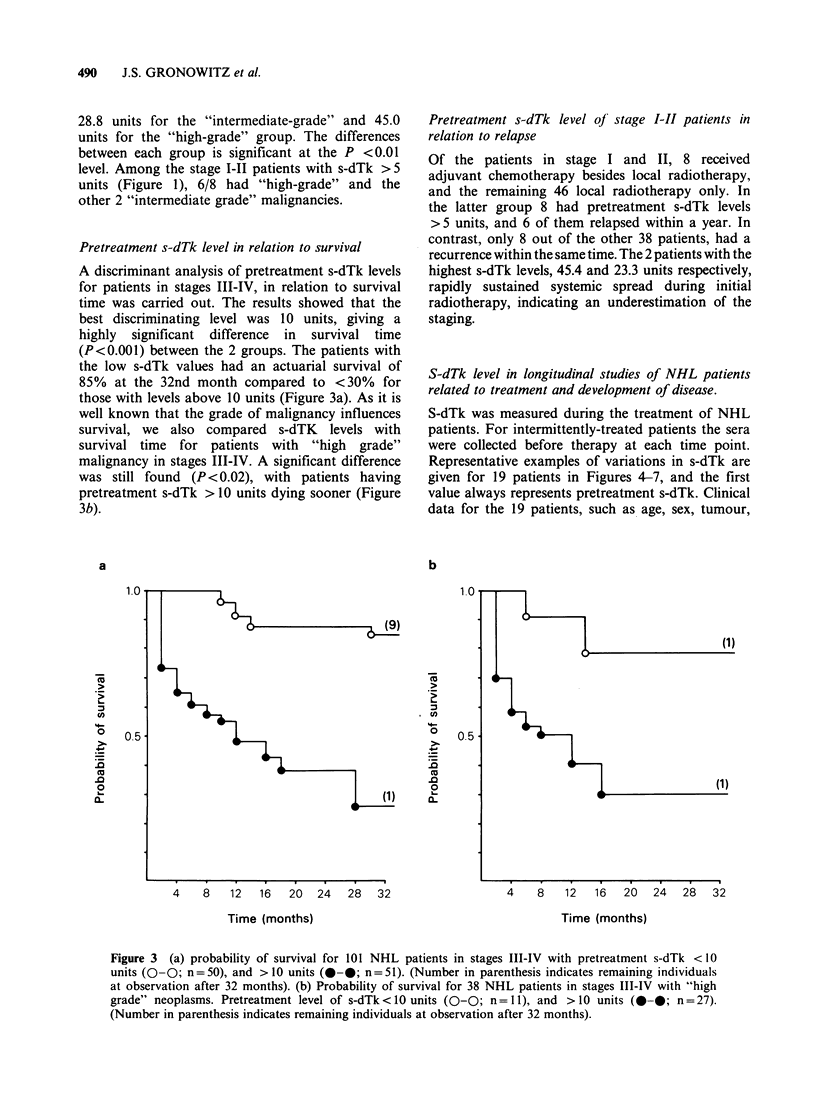

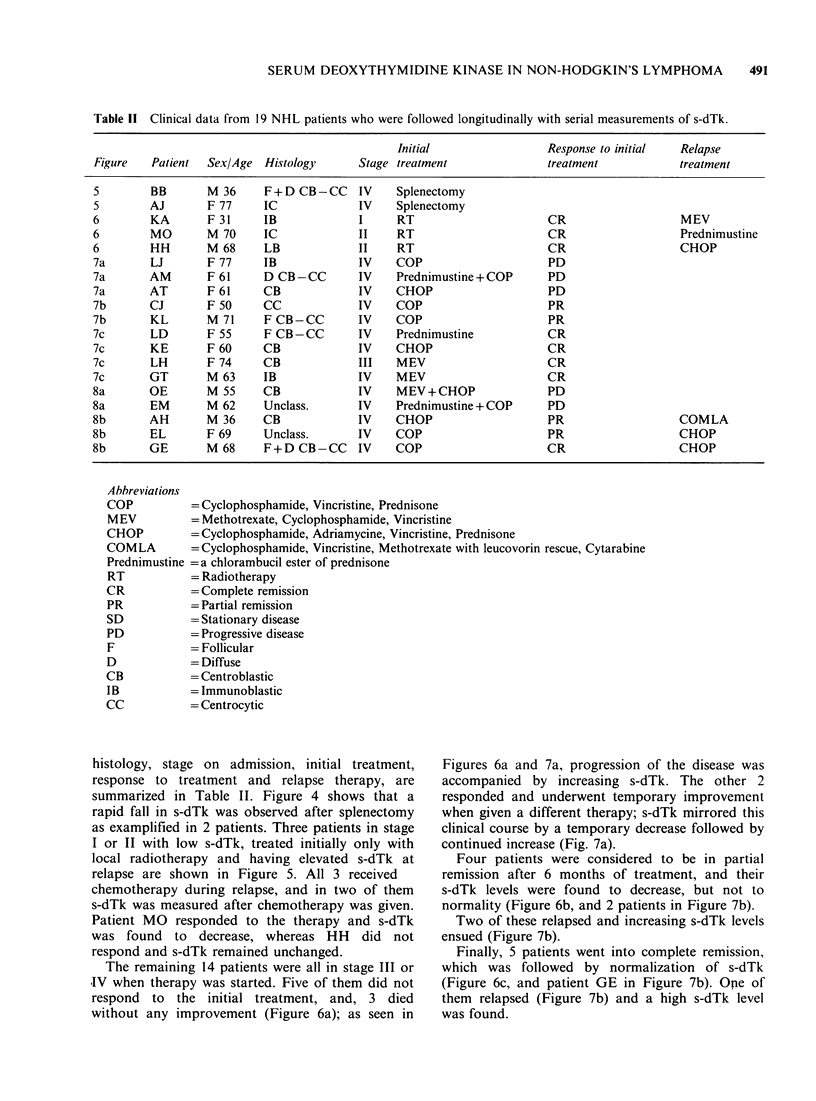

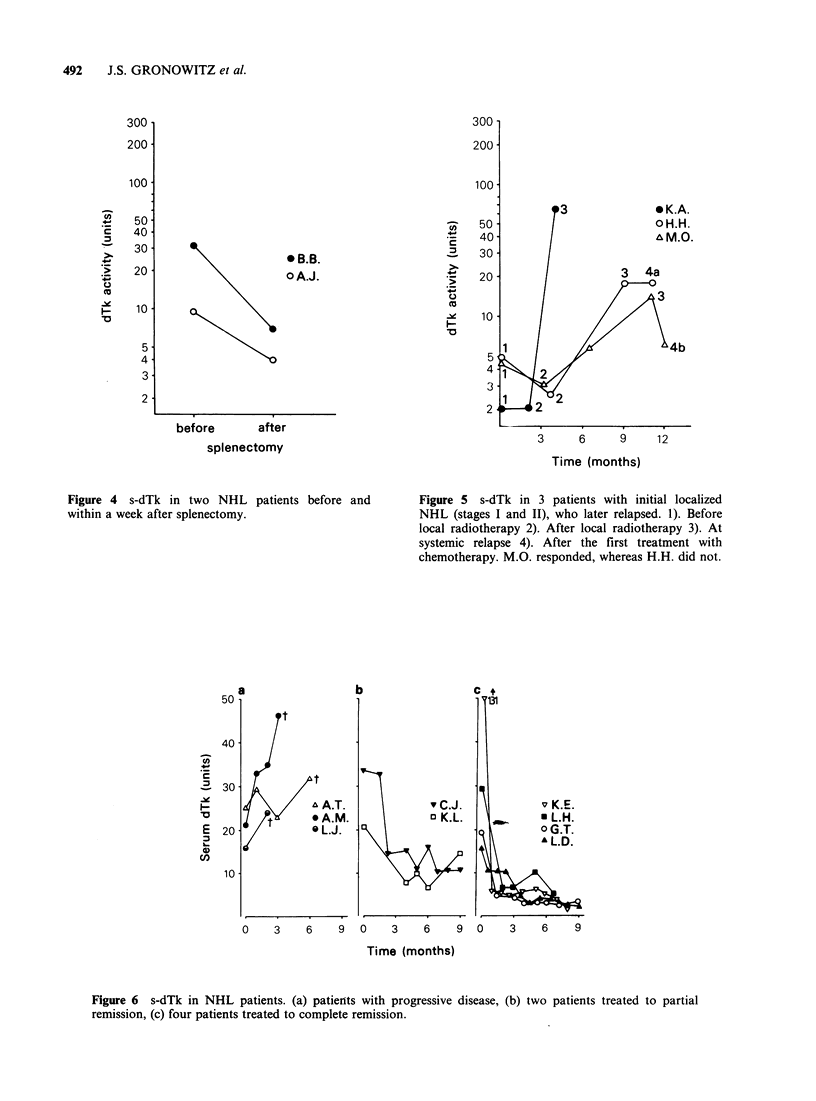

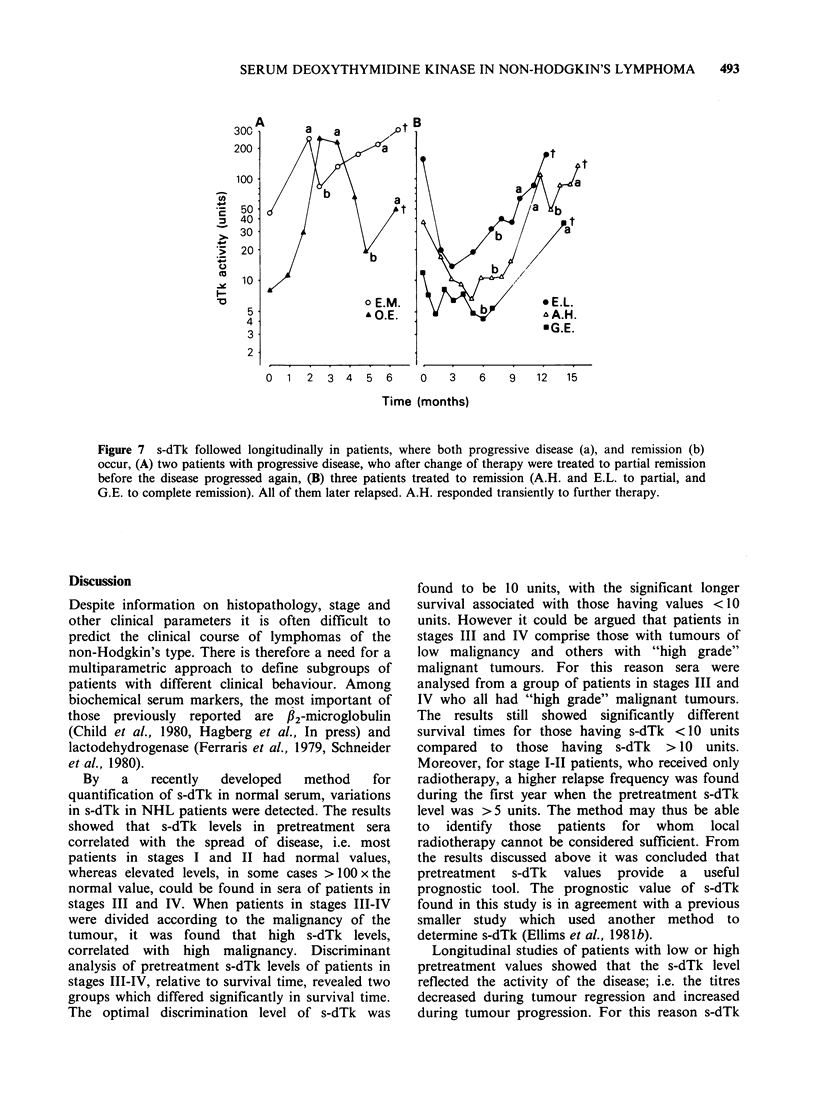

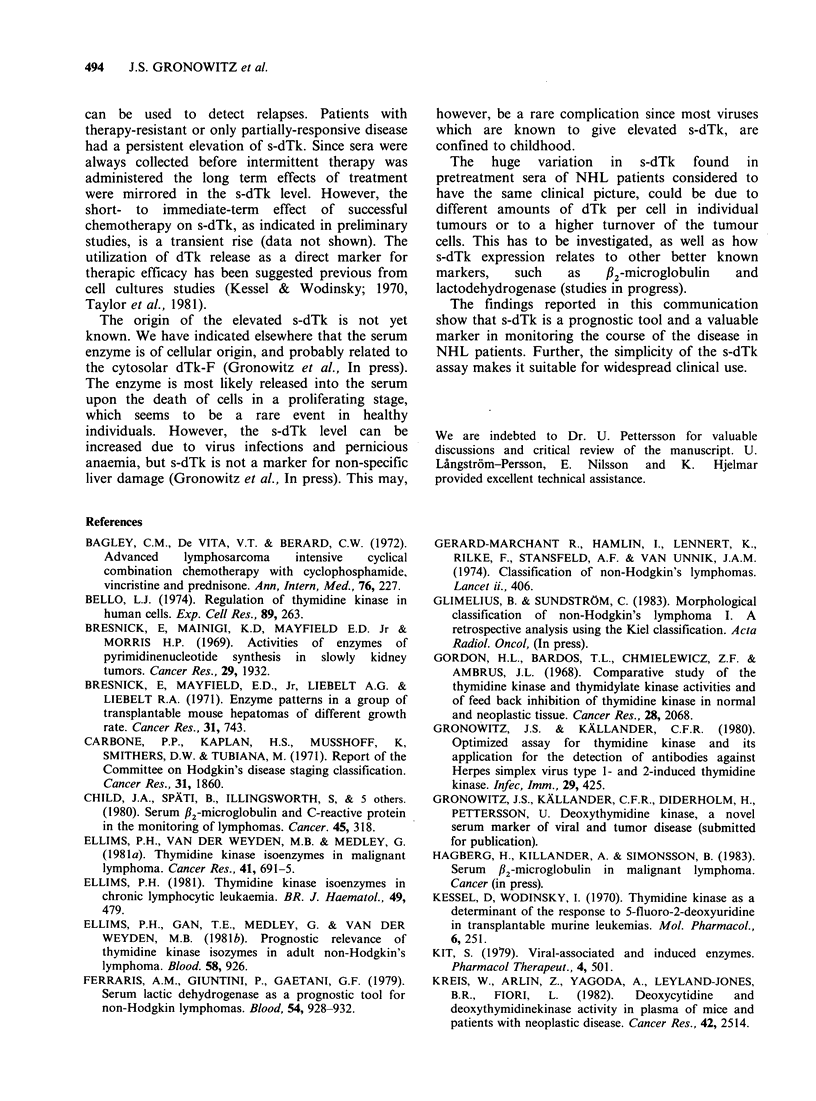

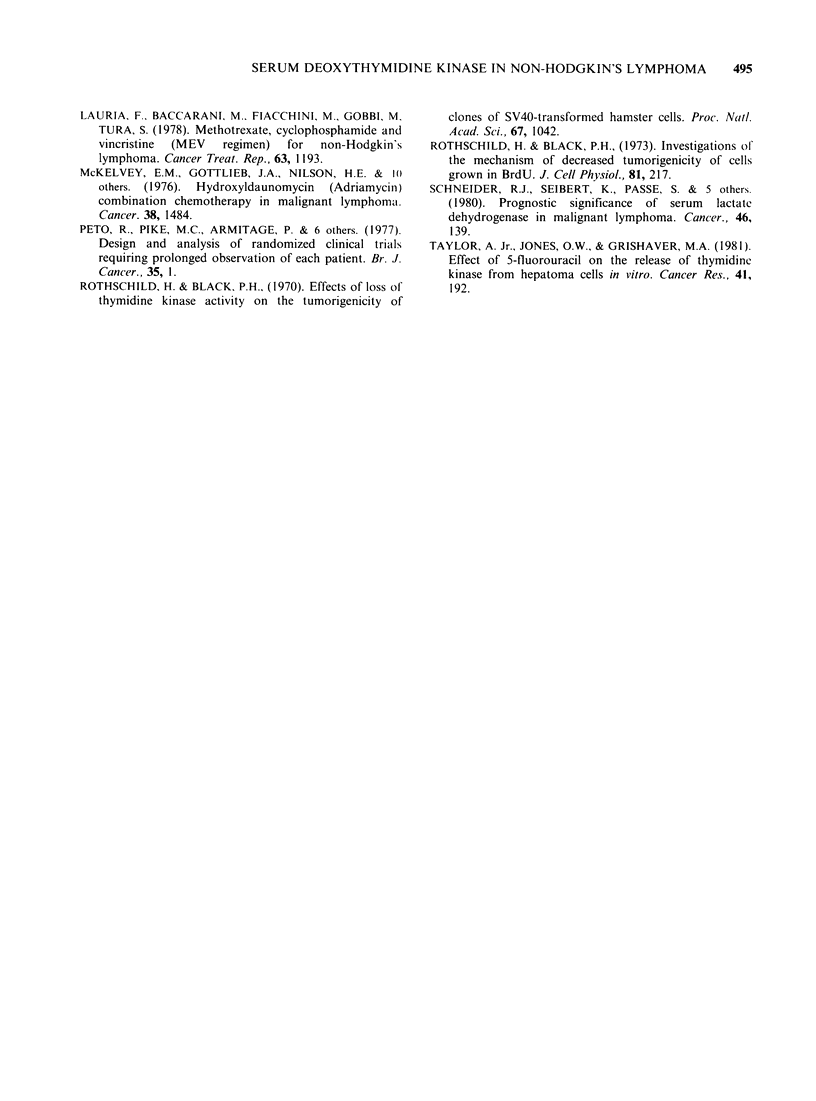


## References

[OCR_00966] Bagley C. M., Devita V. T., Berard C. W., Canellos G. P. (1972). Advanced lymphosarcoma: intensive cyclical combination chemotherapy with cyclophosphamide, vincristine, and prednisone.. Ann Intern Med.

[OCR_00972] Bello L. J. (1974). Regulation of thymidine kinase synthesis in human cells.. Exp Cell Res.

[OCR_00976] Bresnick E., Mainigi K. D., Mayfield E. D., Morris H. P. (1969). Activities of enzymes of pyrimidine nucleotide synthesis in slowly growing kidney tumors.. Cancer Res.

[OCR_00982] Bresnick E., Mayfield E. D., Liebelt A. G., Liebelt R. A. (1971). Enzyme patterns in a group of transplantable mouse hepatomas of different growth rates.. Cancer Res.

[OCR_00988] Carbone P. P., Kaplan H. S., Musshoff K., Smithers D. W., Tubiana M. (1971). Report of the Committee on Hodgkin's Disease Staging Classification.. Cancer Res.

[OCR_00994] Child J. A., Spati B., Illingworth S., Barnard D., Corbett S., Simmons A. V., Stone J., Worthy T. S., Cooper E. H. (1980). Serum beta 2 microglobulin and C-reactive protein in the monitoring of lymphomas: findings in a multicenter study and experience in selected patients.. Cancer.

[OCR_01009] Ellims P. H., Eng Gan T., Medley G., Van Der Weyden M. B. (1981). Prognostic relevance of thymidine kinase isozymes in adult non-Hodgkin's lymphoma.. Blood.

[OCR_01004] Ellims P. H., Gan T. E., Van der Weyden M. B. (1981). Thymidine kinase isoenzymes in chronic lymphocytic leukaemia.. Br J Haematol.

[OCR_01015] Ferraris A. M., Giuntini P., Gaetani G. F. (1979). Serum lactic dehydrogenase as a prognostic tool for non-Hodgkin lymphomas.. Blood.

[OCR_01032] Gordon H. L., Bardos T. J., Chmielewicz Z. F., Ambrus J. L. (1968). Comparative study of the thymidine kinase and thymidylate kinase activities and of the feedbach inhibition of thymidine kinase in normal and neoplastic human tissue.. Cancer Res.

[OCR_01043] Gronowitz J. S., Källander C. F. (1980). Optimized assay for thymidine kinase and its application to the detection of antibodies against herpes simplex virus type 1- and 2-induced thymidine kinase.. Infect Immun.

[OCR_01057] Kessel D., Wodinsky I. (1970). Thymidine kinase as a determinant of the response to 5-fluoro-2'-deoxyuridine in transplantable murine leukemias.. Mol Pharmacol.

[OCR_01063] Kit S. (1979). Viral-associated and induced enzymes.. Pharmacol Ther B.

[OCR_01067] Kreis W., Arlin Z., Yagoda A., Leyland-Jones B. R., Fiori L. (1982). Deoxycytidine and deoxythymidine kinase activities in plasma of mice and patients with neoplastic disease.. Cancer Res.

[OCR_01077] Lauria F., Baccarani M., Fiacchini M., Frezza R., Gobbi M., Tura S. (1978). 5-year survey of methotrexate, cyclophosphamide (Endoxan), and vincristine (MEV) therapy for advanced non-Hodgkin's lymphomas.. Cancer Treat Rep.

[OCR_01081] McKelvey E. M., Gottlieb J. A., Wilson H. E., Haut A., Talley R. W., Stephens R., Lane M., Gamble J. F., Jones S. E., Grozea P. N. (1976). Hydroxyldaunomycin (Adriamycin) combination chemotherapy in malignant lymphoma.. Cancer.

[OCR_01095] Rothschild H., Black P. H. (1970). Effect of loss of thymidine kinase activity on the tumorigenicity of clones of SV40-transformed hamster cells.. Proc Natl Acad Sci U S A.

[OCR_01100] Rothschild H., Black P. H. (1973). Investigations of the mechanism of decreased tumorigenicity of cells grown in BrdU.. J Cell Physiol.

[OCR_01105] Schneider R. J., Seibert K., Passe S., Little C., Gee T., Lee B. J., Miké V., Young C. W. (1980). Prognostic significance of serum lactate dehydrogenase in malignant lymphoma.. Cancer.

[OCR_01111] Taylor A., Jones O. W., Grishaver M. A. (1981). Effect of 5-fluorouracil on the release of thymidine kinase from hepatoma cells in vitro.. Cancer Res.

